# Supplementary material for: Satb2 neurons in the parabrachial nucleus mediate taste perception
Source: Nat Commun. 2021 Jan 11;12:224. doi: 10.1038/s41467-020-20100-8 (PMC7801645; doi:10.1038/s41467-020-20100-8)
Supplement: Supplementary file 1 — Supplementary Information [file 41467_2020_20100_MOESM1_ESM.pdf]

Supplementary Figure 1

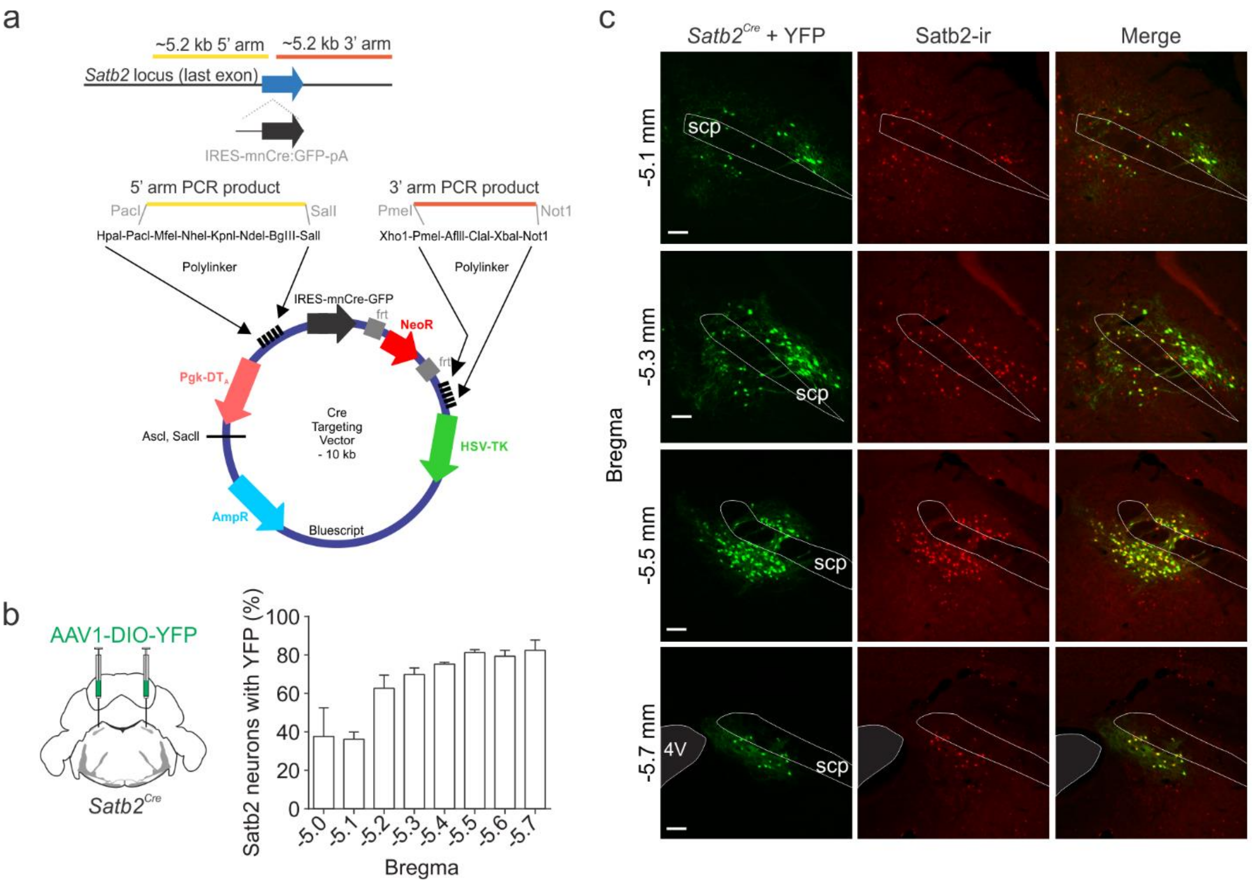

Supplementary Figure 1: Generation of *Satb2^{Cre}* mouse line. a, Diagram for generation of the *Satb2^{Cre}* mouse line showing: Top, the last exon of the *Satb2* gene (which has 11 exons), and the location of the IRES-Cre insert. Bottom, the targeting vector. **b**, AAV1-DIO-YFP injections into PBN of *Satb2*^{Cre} mice and quantification of YFP expression in *Satb2* neurons across the rostal-caudal axis of the PBN (n=3). **c,** Representative images from brains counted in panel b of immunostaining showing colocalization of YFP in *Satb2*^{Cre} in neurons (green) with Satb2-immunoreactivity (red) (n=3). Scale bar, 100 µm. scp, superior cerebellar peduncle. 4V, fourth ventricle. Data are presented as mean ± SEM. Source data are provided as a Source Data file.

Supplementary Figure 2

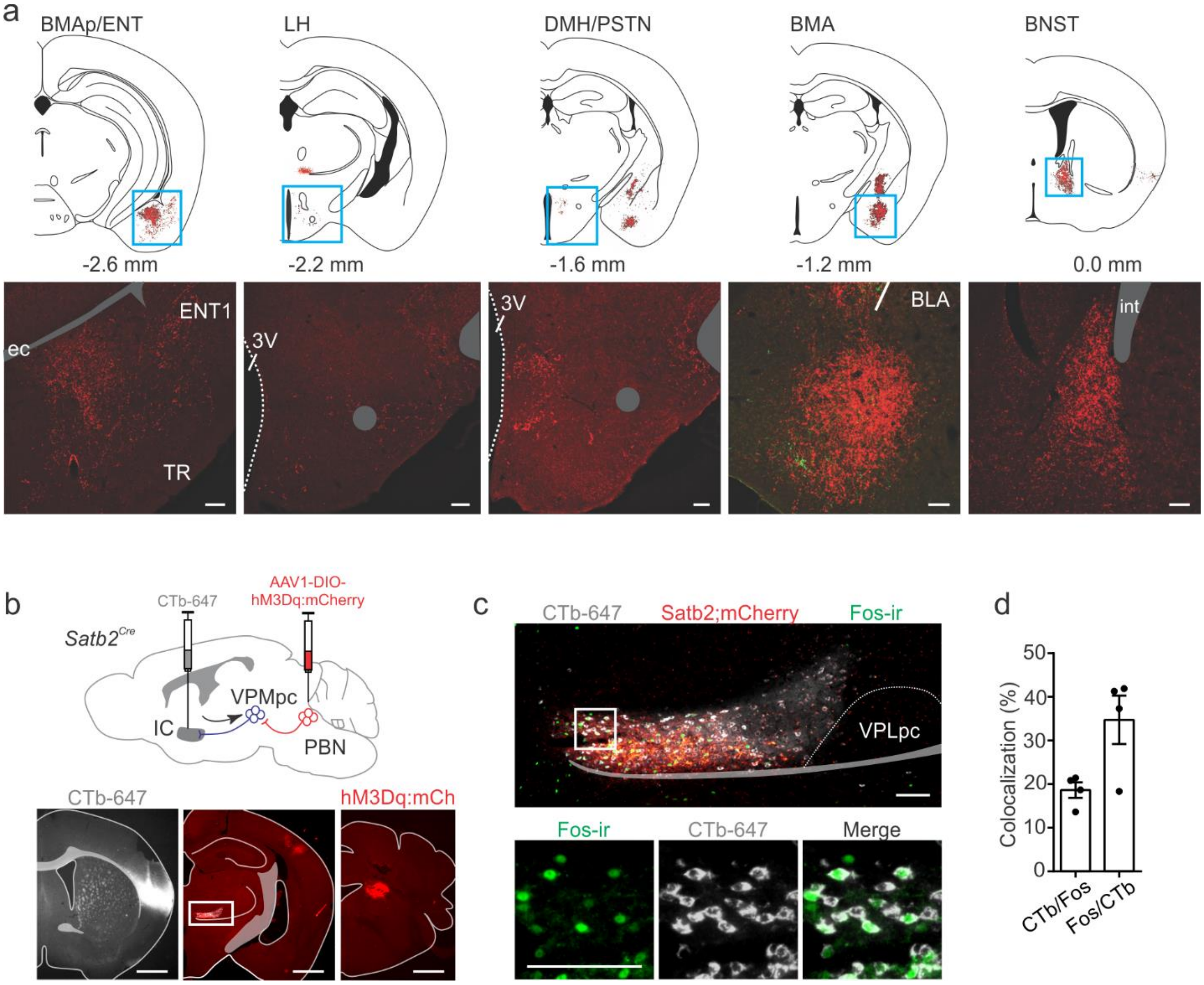

Supplementary Figure 2. Downstream targets of Satb2^{PBN} neurons. a, Extension of Fig. 1b, where AAV1-DIO-Synaptophysin:mCherry was injected unilaterally into the PBN of *Satb2^{Cre}* mice. Diagram of projections from Satb2 neurons in the PBN with images of regions highlighted in blue. (n=3 mice). Scale bar, 100 µm. **b**, Injection of the retrograde tracer cholera-toxin B subunit conjugated to Alexa Fluor 647 (CTb-647) into the IC and AAV1-DIO-hM3Dq:mCherry into the PBN of *Satb2^{Cre}* mice (n=4 mice). Scale bar, 1 mm. **c**, Image of the VPMpc showing expression of CTb-647 from the IC, hM3Dq:mCherry projections from Satb2 neurons in the PBN, and Fos induced by activating hM3Dq:mCherry with CNO. Scale bars, 100 µm. **d**, Quantification of Fos and CTb-647 expression. Data are presented as mean ± s.e.m. BLA, basolateral amygdala; BMA, basomedial amygdala; BNST, bed nucleus of stria terminalis; CeM, central medial nucleus of the amygdala; ec, external capsule; ENT1, entorhinal cortex; IC, insular cortex; int, internal capsule; LH, lateral hypothalamus; PSTN, parasubthalamic nucleus; Scp, superior cerebellar peduncle; TR, postpiriform transition area; VPMpc, parvicellular part of ventral posteriomedial thalamus; VPLpc, ventral posterolateral thalamus, parvicellular part. 3V, third ventricle. Data are presented as mean ± SEM. Source data are provided as a Source Data file.

Supplementary Figure 3

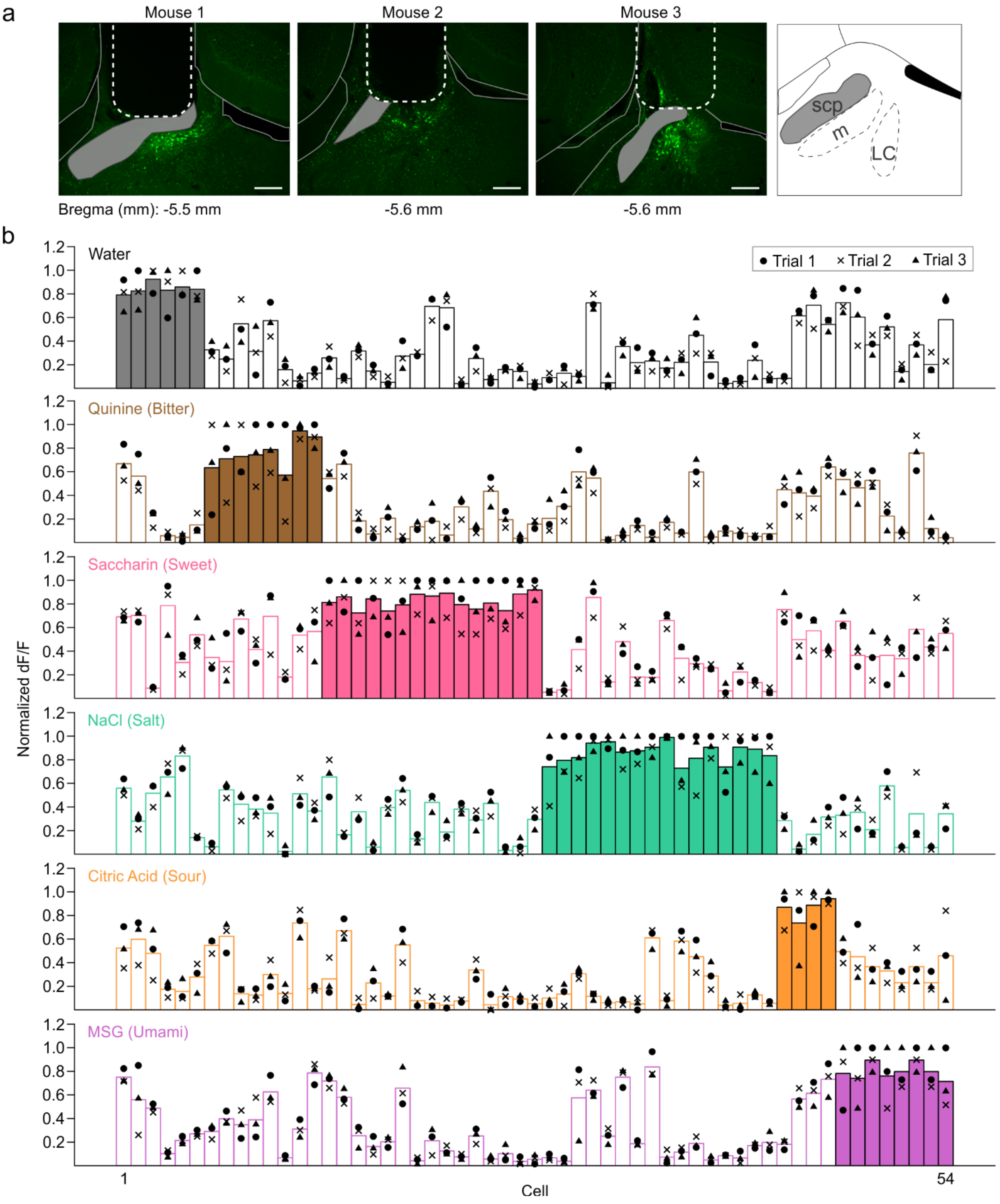

Supplementary Figure 3. Satb2 neurons respond to complex taste stimuli but not temperature or odor. a, Representative images of Satb2 neurons expressing GCaMP6s with approximate lens placement from each of the 3 mice used for imaging experiments (dotted lines). Scale bar, 200 µm; scp, superior

cerebellar peduncle. m, medial parabrachial nucleus. LC, locus coeruleus. **b**, Expanded data from Fig 2e showing average responses of individual Satb2 neurons to different tastants across three trials with symbols representing responses from each trial. Source data are provided as a Source Data file.

Supplementary Figure 4

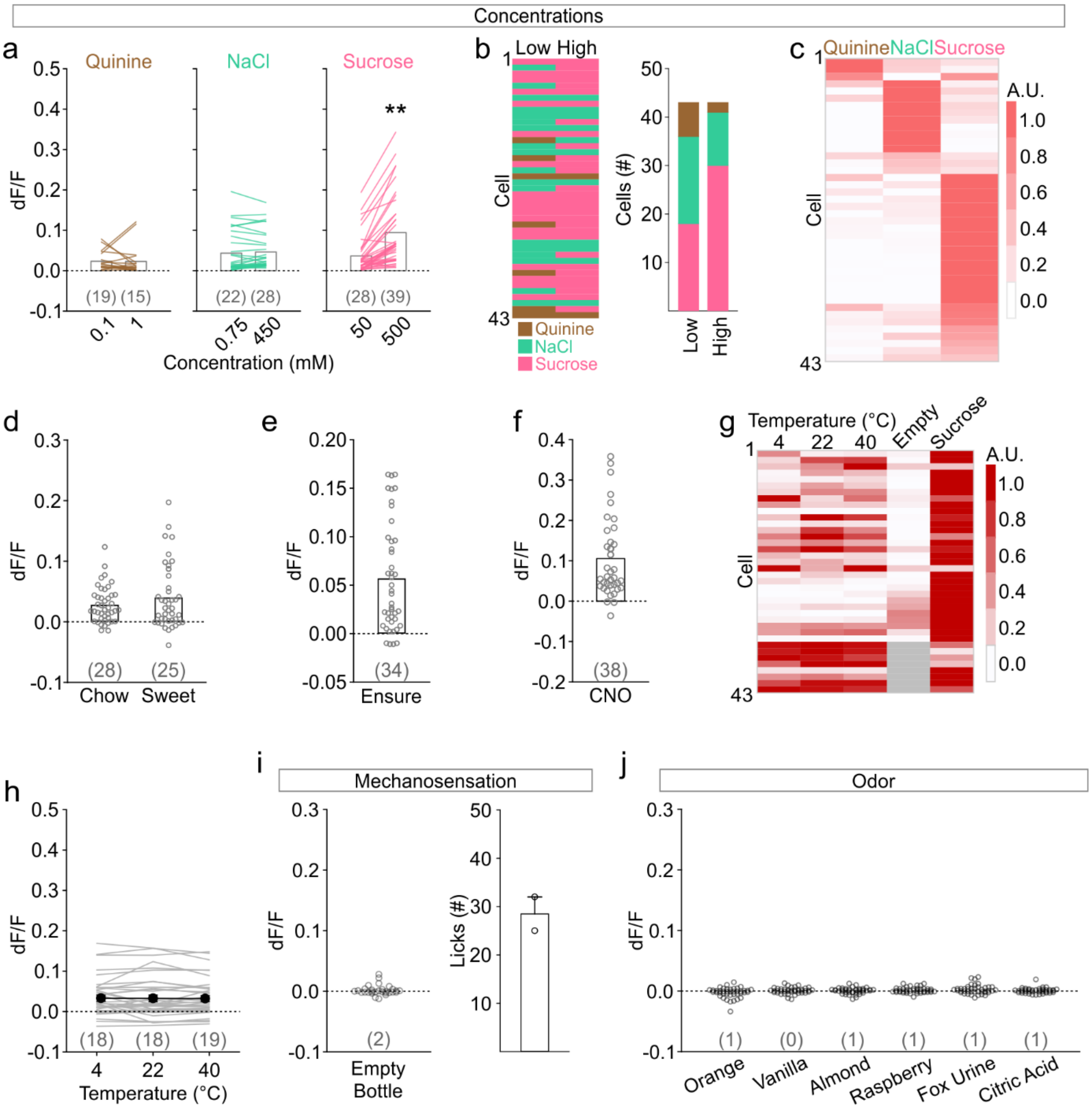

Supplementary Figure 4. Satb2 neurons respond to complex taste stimuli but not temperature or odor. a, Responses of individual Satb2 neurons to licking high and low concentrations of quinine, NaCl, and sucrose, normalized to the number of licks for each taste, data combined from 3 mice. For each taste, only neurons with a significant response at either concentration (>3 SD) were included in the analysis. There was a significant increase in net average response of Satb2 neurons to a high concentration of sucrose, but not quinine or NaCl (Quinine: Wilcoxon signed rank test, n=19 cells from 3 mice, t(18)=-0.150, *P*=0.883; NaCl: paired two—tailed t-test, n=31 cells from 3 mice t(30)=-1.234, *P*=0.227; Sucrose: Wilcoxon signed rank test, n=39 cells from 3 mice, Z=5.247, *P*<0.001). **b**, The best response stimulus for each cell at low and high concentrations. **c**, The product of the normalized responses between low and high concentrations of each cell. **d-f,** Net average responses of Satb2 neurons to solid food (**d**), a liquid food (**e**), and an injection of CNO to activate hM3Dq (**f**). **g**, Average

normalized responses to licking for water at different temperatures or an empty bottle compared to sucrose. **h-j**, There were no significant responses of Satb2 neurons during licking of water at different temperatures (Friedman test, $X^2(2)=4.421$ P=0.11) (**h**) an empty bottle (**i**), normalized to number of licks, and odors presented on a cotton applicator (**j**). Numbers in parenthesis represent number of cells where the net average response >3 SD from baseline *$P < 0.05$, ***$P < 0.001$. Data are presented as mean ± SEM. Source data are provided as a Source Data file.

Supplementary Figure 5

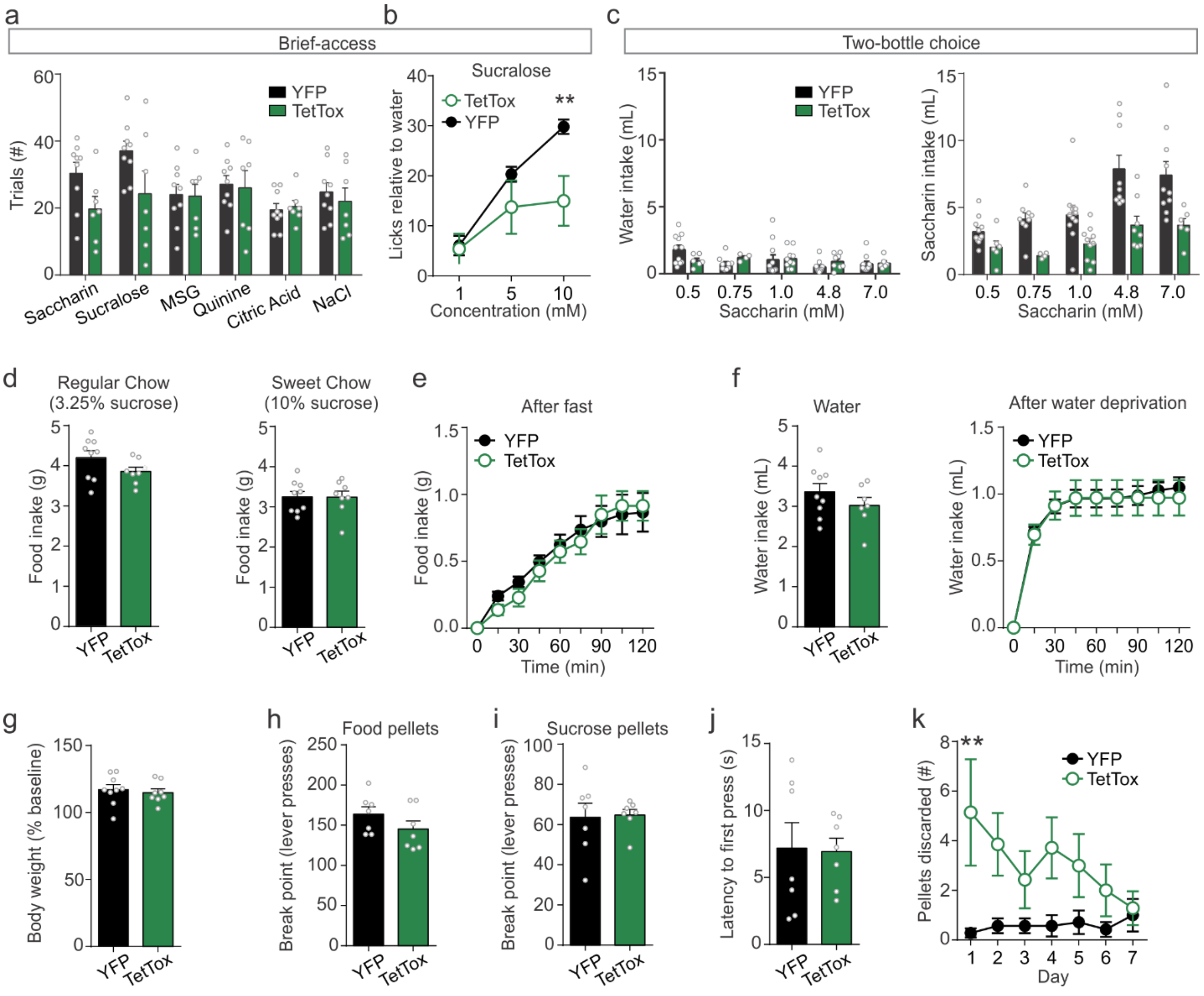

Supplementary Figure 5. Inactivation of Satb2 neurons has no effect on food or water intake. a, There were no significant differences in number of trials completed during brief-access taste tests in Figure 3 between TetTox and YFP mice (YFP n=9, TetTox n=7; multiple t-tests per row with Holm-Sidak correction for multiple comparisons). **b**, TetTox-expressing mice licked less for sucralose, an alternative sweetener, than control mice (YFP n=9, TetTox n=6; two-way RM ANOVA, interaction $F_{(2,26)}$=8.871, *P*=0.0012). **c**, Volume of water and saccharin intake in two-bottle choice tests shown in Figure 3. TetTox-expressing mice drank less saccharin than YFP control mice (YFP n=9-11, TetTox n=4-10; two-way ANOVA; water: group effect $F_{(1, 74)}$=0.0424, *P*=0.8374; saccharin group effect $F_{(1, 74)}$=37.02, *P*=4.73E-08). Exact n per group are provided in Supplementary Table 1. **d**, Mice consumed comparable amounts of regular chow and sweet chow over 24-h (YFP n=9, TetTox n=8, two-tailed unpaired t-test; regular chow: t(15)=1.67, *P*=0.1157; sweet chow: t(15)=0.0441, *P*=0.9654). **e**, After a 16-h fast, TetTox and YFP mice ate a similar amount of sweet chow during re-feeding (YFP n=5, TetTox n=5; two-way RM ANOVA, interaction $F_{(8,64)}$=0.7556, *P*=0.6426). **f**, TetTox and YFP-injected mice drank similar amounts of water over 24-h and following 16-h water deprivation (24-h: YFP n=9, TetTox n=7, two-tailed unpaired t-test, t(14)=1.136, *P*=0.2751; water deprivation: YFP n=8, TetTox n=6; two-way RM ANOVA, $F_{(8,96)}$=0.255, *P*=0.9784). **g**, There was no difference in body weight between the two groups 12-wk post-surgery (YFP n=9, TetTox

n=10; two-tailed unpaired t-test, t(15)=0.4845, *P*=0.635). **h**, In a progressive ratio task, hungry mice from both YFP and TetTox groups reached a comparable break-point for food pellets, averaged across 7 trials (n=7 mice/group, two-tailed unpaired t-test, t(12)=1.38, *P*=0.1927). **i-j**, In *ad libitum* fed mice, there was no difference in the break-point for sucrose pellets (**i**) or latency to the first lever press between groups (**j**) (sucrose: 7 trial average, Mann-Whitney test, U=21, *P*=0.6894; latency: two-tailed unpaired t-test, t(12)=0.117, *P*=0.9088). **k**, TetTox-expressing mice discarded more pellets in initial sessions than control mice (two-way RM ANOVA, group effect $F_{(1, 84)}$=22.41, *P*=8.83E-06). Post-hoc analyses were done with Holm-Sidak's multiple comparison test with *$P < 0.05$, **$P < 0.01$, ****$P < 0.0001$. Data are presented as mean ± SEM. Source data are provided as a Source Data file.

Supplementary Figure 6

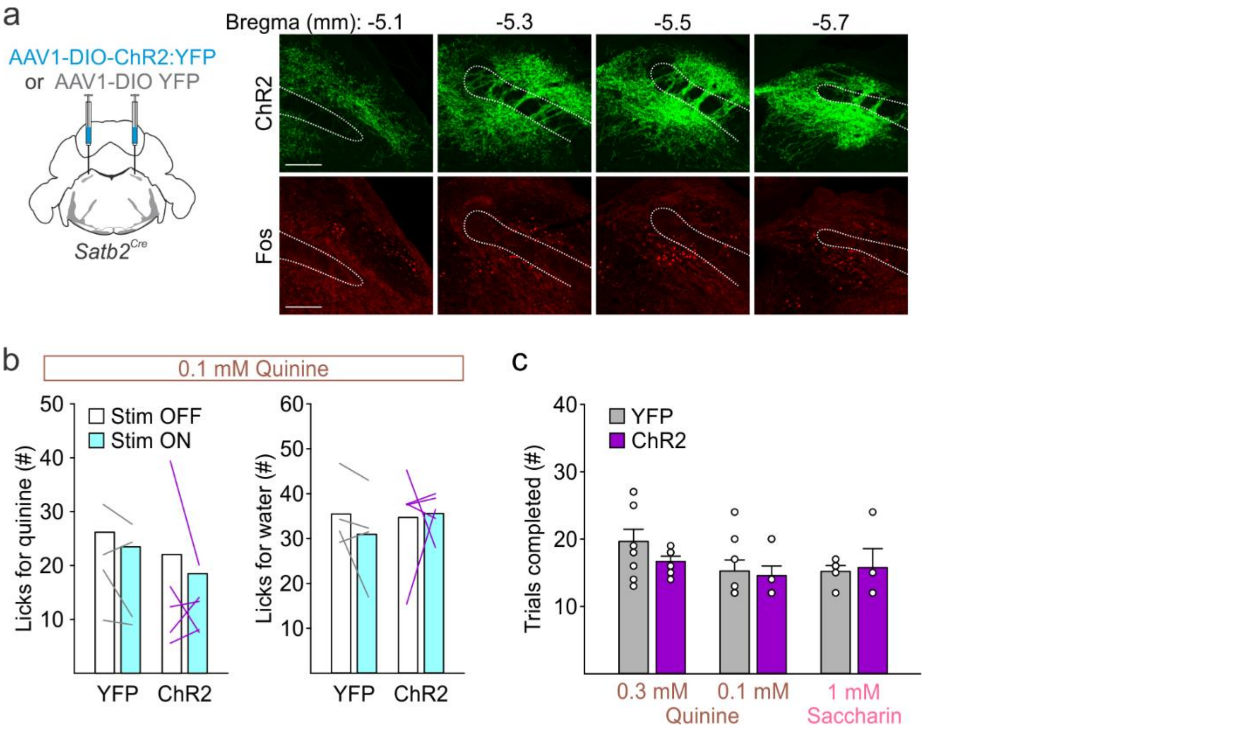

Supplementary Figure 6. Photostimulation induces Fos in Sat2 neurons throughout the PBN. **a**, Bilateral injections of AAV1-DIO-ChR2:YFP or AAV1-DIO-YFP into the PBN of *Satb2*^{Cre} mice and representative image of viral expression and Fos induction throughout the PBN following photostimulation. Similar levels of Fos expression were seen in n=7 mice. Scale bar, 100 µm. **b**, Photostimulation had no effect on licking for 0.1 mM quinine solution or water (one-way RM ANOVA, quinine: interaction $F_{(3,10)}$=0.443, *P*=0.727; water: interaction $F_{(3,10)}$=0.227, *P*=0.875). **c**, There were no significant differences in the number of trials completed in the brief-access taste tests from Figure 5 between ChR2 and YFP mice (0.3 mM quinine: Welch's two-tailed t-test, t(13)=1.295, *P*=0.218; 0.1 mM quinine: Student's two-tailed t-test, t(7)=-0.185, *P*=0.843; 1 mM saccharin: Mann-Whitney U=26, *P*=0.867). Data are presented as mean ± SEM. Source data are provided as a Source Data file.

Supplementary Figure 7

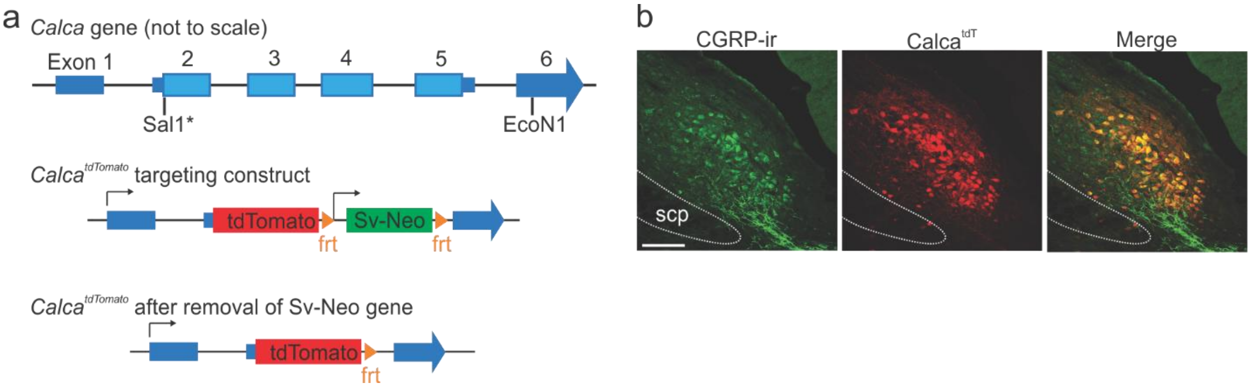

Supplementary Figure 7. Generation of *Calca*^{tdT} mouse line. a, Diagram for generation of the *Calca*^{tdT} mouse line showing: Top, the *Calca* gene and key restriction enzyme sites used for cloning. Middle, the targeting construct (inserted in the same vector shown in Supplementary Fig 1a). Bottom, the *Calca* locus after recombination and removal of the frt-flanked SV-Neo selection gene. **b**, tdTomato expression in the PBN of a *Calca*^{tdT} mouse (red), with immunostaining showing co-localization with CGRP (green) (n=2).

Supplementary Table 1

	YFP (n)	TetTox (n)
Fig 3f: Bitter, 0.1	13	8
Fig 3f: Bitter, 0.3	10	8
Fig 3f: Bitter, 0.5	8	10
Fig 3f: Bitter, 0.7	10	7
Fig 3f: NaCl, 75	7	6
Fig 3f: NaCl, 150	9	7
Fig 3f: NaCl, 300	9	8
Fig 3f: NaCl, 450	13	11
Fig 3f; Citric Acid, 5	11	7
Fig 3f; Citric Acid, 10	14	11
Fig 3f; Citric Acid, 20	11	7
Fig 3f; Citric Acid, 30	9	8
Fig 3g; Saccharin, 0.5	11	6
Fig 3g; Saccharin, 0.75	8	4
Fig 3g; Saccharin, 1.0	10	10
Fig 3g; Saccharin, 4.8	9	8
Fig 3g; Saccharin, 7.0	10	6
Fig 3g; MSG	5	5
Fig 3g; Sucrose	5	5
	YFP (n)	TetTox (n)
Supplementary Fig 5c: 0.5 mM	12	6
Supplementary Fig 5c: 0.75 mM	9	4
Supplementary Fig 5c: 1.0 mM	11	10
Supplementary Fig 5c: 4.8 mM	9	8
Supplementary Fig 5c: 7.0 mM	10	6

Supplementary Table 1: Animal numbers for Fig3 f-g, Supplementary Fig 5c.
