# Supplementary material for: Satb2 neurons in the parabrachial nucleus mediate taste perception
Source: Nat Commun. 2021 Jan 11;12:224. doi: 10.1038/s41467-020-20100-8 (PMC7801645; doi:10.1038/s41467-020-20100-8)
Supplement: Supplementary file 4 — Reporting Summary [file 41467_2020_20100_MOESM4_ESM.pdf]

nature research

Corresponding author(s): Richard D. Palmiter

Last updated by author(s): Oct 20, 2020

Reporting Summary

Nature Research wishes to improve the reproducibility of the work that we publish. This form provides structure for consistency and transparency in reporting. For further information on Nature Research policies, see our Editorial Policies and the Editorial Policy Checklist.

Statistics

For all statistical analyses, confirm that the following items are present in the figure legend, table legend, main text, or Methods section.

n/a	Confirmed	
☐	☒	The exact sample size (*n*) for each experimental group/condition, given as a discrete number and unit of measurement
☐	☒	A statement on whether measurements were taken from distinct samples or whether the same sample was measured repeatedly
☐	☒	The statistical test(s) used AND whether they are one- or two-sided<br>*Only common tests should be described solely by name; describe more complex techniques in the Methods section.*
☐	☒	A description of all covariates tested
☐	☒	A description of any assumptions or corrections, such as tests of normality and adjustment for multiple comparisons
☐	☒	A full description of the statistical parameters including central tendency (e.g. means) or other basic estimates (e.g. regression coefficient) AND variation (e.g. standard deviation) or associated estimates of uncertainty (e.g. confidence intervals)
☐	☒	For null hypothesis testing, the test statistic (e.g. *F*, *t*, *r*) with confidence intervals, effect sizes, degrees of freedom and *P* value noted<br>*Give P values as exact values whenever suitable.*
☒	☐	For Bayesian analysis, information on the choice of priors and Markov chain Monte Carlo settings
☒	☐	For hierarchical and complex designs, identification of the appropriate level for tests and full reporting of outcomes
☒	☐	Estimates of effect sizes (e.g. Cohen's *d*, Pearson's *r*), indicating how they were calculated

Our web collection on statistics for biologists contains articles on many of the points above.

Software and code

Policy information about availability of computer code

Data collection	Calcium imaging experiments were collected with nVista 2 software 1.2 (Inscopix) software, licking data were collected with pClamp 11.0 software (Molecular Devices).
Data analysis	Calcium imaging data was analyzed with Inscopix Data Processing Software 1.2 (Inscopix), licking data were analyzed with pClamp 11.0 (Molecular Devices), behavioral data were analyzed with Excel (Microsoft Office), Prism 6.0 (GraphPad Software) and SigmaPlot 14.0 (Systat Software) was used for all statistical analysis.

For manuscripts utilizing custom algorithms or software that are central to the research but not yet described in published literature, software must be made available to editors and reviewers. We strongly encourage code deposition in a community repository (e.g. GitHub). See the Nature Research guidelines for submitting code & software for further information.

Data

Policy information about availability of data

All manuscripts must include a data availability statement. This statement should provide the following information, where applicable:

- Accession codes, unique identifiers, or web links for publicly available datasets
- A list of figures that have associated raw data
- A description of any restrictions on data availability

Source data are provided with the paper as a Source Data file

Field-specific reporting

Please select the one below that is the best fit for your research. If you are not sure, read the appropriate sections before making your selection.

☒ Life sciences ☐ Behavioural & social sciences ☐ Ecological, evolutionary & environmental sciences

For a reference copy of the document with all sections, see nature.com/documents/nr-reporting-summary-flat.pdf

Life sciences study design

All studies must disclose on these points even when the disclosure is negative.

Sample size	No statistical analyses were done to pre-determine sample size. Group sizes were chosen based on previous studies performed in the lab, some unpublished as well as published (Jarvie & Palmiter, Nat Neuro, 2017)(Chen at el., Neuron, 2018).
Data exclusions	Animals that received viral injections that missed the target (less than 10 fluorescent cells visible/section) were excluded from analysis. These criteria were pre-established.
Replication	All attempts at replication of behavioral studies were succcessful, and the data reported for these experiments were collected from multiple independent cohorts. This included anywhere from 2-4 independently run groups of mice per experiment.
Randomization	Male and female mice from the same litter were split randomly between control and experimental groups, roughly half and half per group
Blinding	Investigators were blinded to group allocation during data collection during brief-access tests; for all other tests and data analysis the investigators were not officially blinded.

Reporting for specific materials, systems and methods

We require information from authors about some types of materials, experimental systems and methods used in many studies. Here, indicate whether each material, system or method listed is relevant to your study. If you are not sure if a list item applies to your research, read the appropriate section before selecting a response.

Materials & experimental systems

n/a	Involved in the study	
☐	☒	Antibodies
☒	☐	Eukaryotic cell lines
☒	☐	Palaeontology and archaeology
☐	☒	Animals and other organisms
☒	☐	Human research participants
☒	☐	Clinical data
☒	☐	Dual use research of concern

Methods

n/a	Involved in the study	
☒	☐	ChIP-seq
☒	☐	Flow cytometry
☒	☐	MRI-based neuroimaging

Antibodies

Antibodies used

rabbit anti-Satb2 1:2500 (ab34735, Abcam), mouse anti-Satb2 1:1500 (ab34735, Abcam), rabbit anti-Fos 1:2000 (#22505, Cell Signaling; ab190289, Abcam), goat anti-Fos 1:300 (sc-52-G, Santa Cruz), chicken anti-GFP 1:10,000 (ab13970, Abcam), goat anti-CGRP 1:1000 (ab36001, Abcam), rabbit anti-dsRed 1:1000 (632475, Takara).
The following secondary antibodies are all from Jackson Immunoresearch Laboratories:
Alexa-Fluor 488 donkey anti-sheep (713-545-003), Alexa-Fluor 594 donkey anti-sheep (713-585-003), Alexa-Fluor 488 donkey anti-goat (705-545-147), Alexa-Fluor 594 donkey anti-goat (705-585-147), Alexa Fluor 488 donkey anti-rabbit (711-545-152), Alexa Fluor 594 donkey anti-rabbit (711-585-152), or Alexa-Fluor 488 donkey anti-chicken (703-545-155)

Validation

rabbit anti-Satb2 1:2500 (ab34735, Abcam) – validated in mice by Abcam via Western blot, confirmed in this manuscript
mouse anti-Satb2 1:1500 (ab51502, Abcam), validated in mice by Abcam via Western blot, confirmed in this manuscript
rabbit anti-Fos 1:2000 (#22505, Cell Signaling; ab190289, Abcam) – validated in mice by Abcam via WB, ICC, IHC; De Solis, A.J., Baquero, A.F., Bennett, C.M., Grove, K.L. & Zeltser, L.M. Mol. Metab. 5, 189–209 (2016),
goat anti-Fos 1:300 (sc-52-G, Santa Cruz) – validated in previous publication Ma, C.-W. et al. J. Comp. Neurol. 521, 612–625 (2013)
chicken anti-GFP 1:10,000 (ab13970, Abcam) – validated in mice by Abcam via WB, IHC, iCC; Enjin, A. et al. J. Comp. Neurol. 518, 2284–2304 (2010).
goat anti-CGRP 1:1000 (ab36001, Abcam) – validated in previous publication Chen J.Y. et al. Neuron 100, 891-899 (2018)
rabbit anti-dsRed 1:1000 (632475, Takara) – validated in previous publication Bochorishvili, G., Stornetta, R.L., Coates, M.B. & Guyenet, P.G. J. Comp. Neurol. 520, 1047–1061 (2012).

Animals and other organisms

Policy information about studies involving animals; ARRIVE guidelines recommended for reporting animal research

Laboratory animals	Heterozygous CalcaCre 28, Calcatdt, heterozygous and homozygous Satb2Cre or C57Bl/6J (wild-type) mice were used for all experiments, ranging in age from 6-24 weeks. Male and female mice from the same litter were split randomly between control and experimental groups, roughly half and half per group.
Wild animals	No wild animals were used in the study
Field-collected samples	No field collected samples were used in the study
Ethics oversight	All experiments were approved by the Institutional Animals Care and Use Committee at the University of Washington.

Note that full information on the approval of the study protocol must also be provided in the manuscript.
